# Histochemical Analysis of Altered Arachnoid Tissue in Patients With Paroxysmal Trigeminal Neuralgia With Concomitant Continuous Pain

**DOI:** 10.7759/cureus.61502

**Published:** 2024-06-01

**Authors:** Mauro A Segura-Lozano, Octavio Carranza-Rentería, Graciela Velázquez-Delgado, Aarón G Munguía-Rodríguez

**Affiliations:** 1 Neurology, Neurología Segura Medical Center, Hospital Angeles Morelia, Morelia, MEX

**Keywords:** chronic inflammation, arachnoiditis, microvascular cecompression, immunohistochemistry, trigeminal neuralgia

## Abstract

Background

Trigeminal neuralgia (TN) is a craniofacial pain characterized by sudden onset, brief, severe, recurrent shooting pain within one or more branches of the trigeminal nerve (CN V). Based on its clinical presentation, TN may be classified as purely paroxysmal or paroxysmal with concomitant continuous pain (CCP), previously known as typical and atypical, respectively. Microvascular decompression (MVD) surgery for releasing the CN V from a neurovascular conflict is an effective and safe treatment for TN. During MVD of patients manifesting TN with CCP, the involvement of an abnormal arachnoid tissue is a common finding. The etiology and pathophysiology behind the appearance of this tissue are unknown; however, it is more commonly found in this variant of the disease.

Methods

From January 2015 to December 2016, a total of 330 patients diagnosed with TN were evaluated at our clinic. Among them, 31 individuals (9.4%) presented with paroxysmal TN with CCP, with 16 patients (51.6%) undergoing MVD. During surgery, samples of altered arachnoid tissue were collected from five patients and subjected to Hematoxylin-Eosin staining and immunohistochemistry for S100 and CD2

Results

In a long-term follow-up, 80% of patients operated by DMV remains pain free. Analysis of biopsies revealed chronic fibrosis (n=4), hyperplasia of neurothelial cells (n=3), dystrophic calcifications (n=1). Immunohistochemistry was positive for S100 (n=3) and CD20 (n=3) inflammatory markers.

Conclusion

Chronic inflammation in the arachnoid tissue involved in paroxysmal TN with CCP could be a contributor to the pathophysiology of this variant of the disease.

## Introduction

Trigeminal neuralgia (TN) is a neuropathic pain disorder characterized by excruciating pain similar to an electric shock. The pain usually manifests on one side of the face and involves one or more branches of the fifth cranial nerve (CN V). Patients suffering from TN often mention that even innocuous stimuli can trigger severe pain attacks, which significantly affect their quality of life [1]. The disease was classified into three types according to its etiology: classical if the cause of the neuralgia was a neurovascular contact demonstrated through an MRI scan; secondary if neuralgia arises as a clear manifestation of another underlying condition (e.g., tumor compression, multiple sclerosis, malformation, etc.); and idiopathic when the cause of the neuralgia was not identified [2]. Microvascular decompression (MVD) surgery for releasing the CN V from a neurovascular conflict is an effective and safe treatment for classical TN [3].

Two types of clinical presentation are proposed regardless of the etiology: TN with paroxysmal attacks of pain and TN with paroxysmal attacks and concomitant continuous pain (CCP), previously known as typical and atypical TN, respectively [1]. In addition to the sudden episodes of facial pain, patients presenting TN with CCP experience a baseline level of pain that never disappears. This persistent discomfort is frequently described as dull, aching, throbbing, and/or burning, making it challenging to localize to a specific trigger point on the face [2]. The clinical profile of the majority of our patients suffering from TN with CCP includes a long history of pain refractory to various pharmacological alternatives (non-steroidal anti-inflammatory drugs (NSAIDs), antiseizure medications, antidepressants, narcotics) as well as ablative treatments (peripheral and Gasser ganglion blocks) and dental or maxillofacial interventions. Furthermore, some of these patients present pharmacological dependence, anxiety, and depression, with an increased risk of suicide [1,2]. This highlights the importance of prompt diagnosis, treatment, and thorough investigation.

The mechanisms leading to CCP probably differ from those underlying only paroxysmal pain, as suggested by the evidence that pharmacological treatment with sodium channel blockers improves this type of pain less effectively than paroxysmal pain [4]. To date, there is no satisfactory explanation for the mechanisms underlying this type of clinical presentation. Some research on TN is dedicated to exploring previously unconsidered mechanisms underlying this condition. A recent study demonstrated the presence of inflammatory biomarkers in the cerebrospinal fluid (CSF) of patients with TN, specifically tumor necrosis factor β (TNF-β) and TNF-related apoptosis-inducing ligand (TRAIL) [5]. This evidence of inflammation has also been supported by descriptions of an apparent inflammation of the arachnoid and its association with a worse prognosis after MVD in these patients [6]. This association could explain the pain presentation and the evolution of TN with CCP towards the contralateral side of the face. However, much remains to be elucidated about the pathophysiology of this presentation of TN.

The objective of this study was to identify macroscopic and microscopic pathological findings in the altered arachnoid membrane of patients seen in our clinic who underwent MVD to treat TN with CCP, to discuss the possible implications of these findings within the pathophysiology of trigeminal neuralgia and its impact on the immediate and long-term post-surgical outcome.

## Materials and methods

Patients

We conducted a retrospective study of TN cases seen at our clinic between January 2015 and December 2016. During this period, we evaluated 330 patients with TN, among whom 31 (9.4%) were diagnosed with paroxysmal TN with CCP, of which 16 patients (51.6%) underwent MVD surgery. The patients were selected for surgery after a comprehensive preoperative evaluation, during which MRI revealed neurovascular contact. The remaining patients decided not to undergo MVD and continue with pharmacological treatment, underwent alternative surgical interventions at another clinic, or were lost to follow-up. Among operated patients presenting TN with CCP, only five met the inclusion criteria: MRI confirmation of neurovascular conflict, a minimum follow-up period of seven years, and a suitable arachnoiditis sample for histopathological analysis. 

Patient age, sex, affected side, branches involved, and postsurgical follow-up were included in our database. We classified patients as persistent or recurrent based on whether the pain reappeared before or after the first three months post-surgery. Given the poor outcomes post-MVD for patients with TN with CCP [7], surgery was considered successful if there was pain resolution, a decrease in the frequency of pain, or a transition from continuous persistent to episodic pain during the follow-up period.

Surgical technique

The MVD surgery was performed under total intravenous anesthesia (TIVA) in a supine decubitus position. A minimally invasive retrosigmoid craniectomy was made, followed by a durotomy, allowing for the release of CSF and retraction of the cerebellum to access the cerebellopontine cistern. The cisternal segment of the CN V was identified and released from any contact that surrounding vessels or other structures may exert. An appropriate amount of Teflon cotton was inserted to separate the CN V from the offending vessels. The durotomy was closed using aponeurosis and a tight suture. The craniotomy was covered with an absorbable or titanium plate. Closure of the muscle fascia, aponeurosis, and scalp was achieved by suturing each layer. All operations were performed by the same neurosurgeon.

Tissue obtaining and staining

To elucidate the nature of microstructural changes associated with abnormal arachnoid, tissue of approximately 5 mm² were obtained by gently retracting the tissue and cutting it with microscissors. This tissue is routinely removed during MVD when it is involved in the neurovascular conflict. The tissue was then transported in formalin for pathological analysis. Initially, Hematoxylin-Eeosin staining (H&E) was conducted using an automated stainer in our hospital laboratory. Thereafter, immunohistochemical staining for S100 and CD20 was performed on paraffin-embedded sections by using the corresponding monoclonal antibodies (1:100). Detection was carried out via the avidin-biotin-peroxidase complex method, and the sections were counterstained with hematoxylin. Out of 15 specimens, only five were intact and large enough to allow for both pathological analyses to be conducted accurately.

## Results

The mean age of patients was 41.2 years (range, 27-49), with four female patients (80%) and one male patient (20%). The left side was more frequently affected than the right (3:2). The CN V branches involved were V1, V2, V1+V2, and V1+V2+V3 in two cases. The average duration of disease evolution for these patients was 4.1 years (range, 1.5-7). The surgery was successful in 80% of the cases, remaining pain-free in the long-term follow-up (seven years). Only one patient experienced a recurrence of pain in the first month after the surgery (Table 1). Unfortunately, one patient developed TN with the same previous characteristics on the contralateral side; she underwent another DMV five years after the initial intervention. Despite the lower expectations of improvement in patients with TN with CCP [2,7], MVD surgery has yielded positive outcomes, improving the quality of life of our patients during long-term follow-up.

**Table 1:** Summary of the patients' data V1 - ophthalmic branch; V2 - maxillary branch; V3 - mandibular branch; AICA - Anterior-inferior cerebellar artery; SCA - superior cerebellar artery; SPVC - superior petrosal venous complex; * - patient who developed contralateral trigeminal neuralgia

Patient	Age	Sex	Affected side	Distribution of pain	Years of pain	Compressing vessels	Outcome
								1	49	M	R	V1	6	None	Persistent
2	47	F	L	V2	2	SCA	Relief
3	27	F	R	V1, V2	4	AICA, SPVC	Relief*
4	35	F	L	V1, V2, V3	7	SCA, SPVC	Relief
5	48	F	L	V1, V2, V3	1.5	SCA, SPVC	Relief

**Figure 1:**
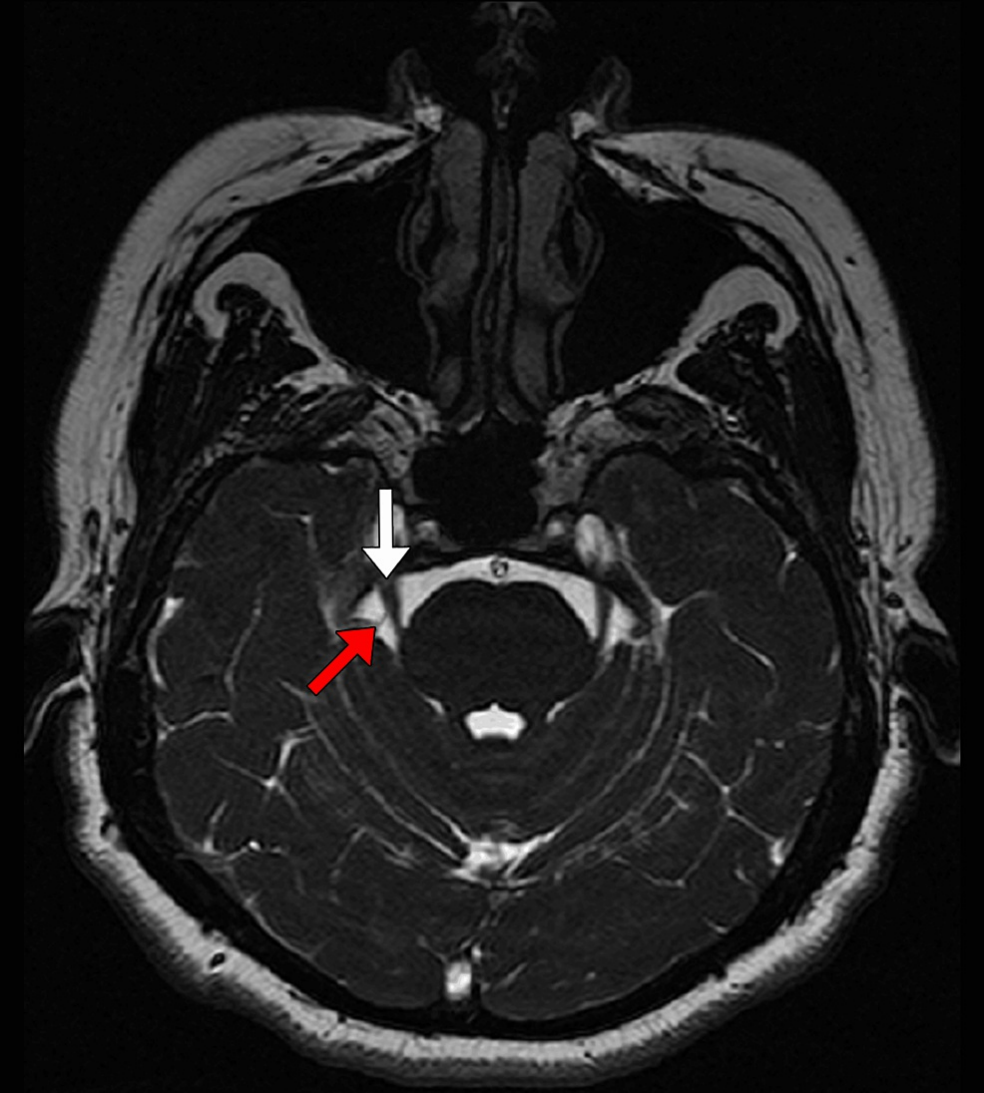
3D-FIESTA MRI showing a false negative for vascular conflict involving the CN V The white arrow indicates the right fifth cranial nerve (CN V) in its cisternal path, and the red arrow indicates a possible culprit vessel. The MRI corresponds to patient one. FIESTA - fast imaging employing steady-state acquisition

The routine 3D-fast imaging employing steady-state acquisition (FIESTA) MRI showed neurovascular contact on CN V in all five cases. However, during the surgical examination of one of the patients, a culprit vessel could not be identified, yielding a false positive result for the MRI (Figure 1). Interestingly, this patient was the one who experienced persistent pain after MVD (Table 1). The reported specificity and sensitivity of MRI in identifying neurovascular conflicts in the CN V are far from reaching 100% [8,9]. Therefore, while the accuracy of imaging studies is improved, the diagnosis of TN with CCP relies mainly on clinical features. Nevertheless, MRI should never be omitted, as it may indicate the presence and location of a neurovascular conflict on the symptomatic side, in addition to being useful for identifying secondary causes of TN or other structural abnormalities in the posterior fossa that may compress the CN V. Currently, for patients without evidence of neurovascular compression on MRI, surgery is not a recommended procedure.

**Figure 2:**
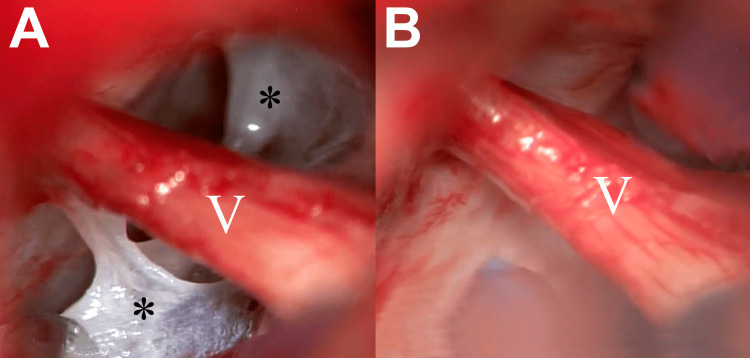
Intraoperative microscopic view during a left microvascular decompression surgery in the cerebellopontine angle A) Thickened arachnoid membranes (*) adhered to neighboring structures and the root of the trigeminal nerve (V) without evident neurovascular conflict. B) Thickened arachnoid removal for nerve release and proper observation of the nerve root. The image corresponds to patient one.

The abnormal arachnoid tissue found during MVD surgery in patients with paroxysmal TN with CCP may be described as thickened membranes with a web-like structure (Figure 2A). We observed that this tissue may adhere either focally or along the nerve root, exhibiting a viscous or fibrous consistency, and varying in severity. The removal of the arachnoid adhesions is imperative to enable a proper observation of the entire nerve root, a critical step in the MVD technique (Figure 2B). This tissue removal is performed under endoscopic or microscopic vision to mitigate the risk of accidentally rupturing or damaging neurovascular structures, such as venules, arterioles, or nerve fillets. Occasionally, arachnoiditis appears capable of compressing the nerve on its own, forming a progressive coalescence that may involve other structures of the trigeminal cistern, causing anatomical alterations that may compress the nerve and complicate its manipulation during surgery.

**Figure 3:**
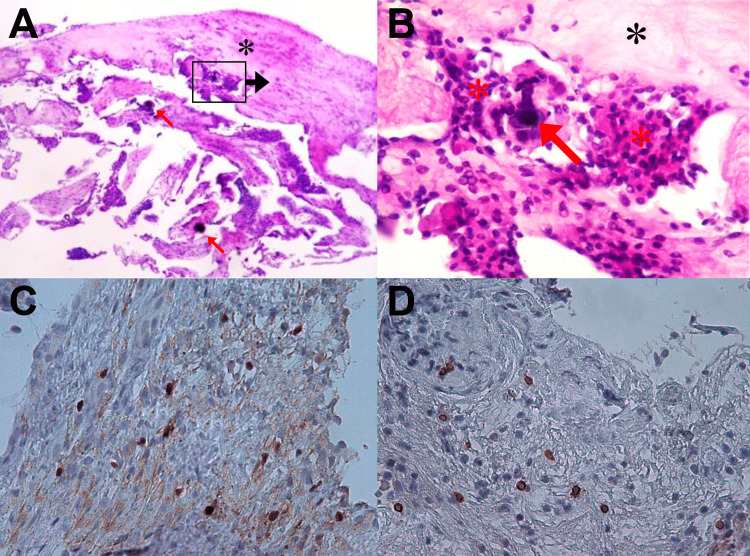
Pathological analysis of abnormal arachnoid tissues. A, B) H&E stain showing dystrophic calcifications (red arrows) associated with chronic fibrosis (black asterisk) and hyperplasia of neurothelial cells (red asterisk). C, D) Immunohistochemical staining for S100 and CD20, respectively. Brown coloration indicates the target cells of each marker. Panels A-C correspond to patient two, while panel D belongs to patient three.

The analysis of arachnoid biopsies after H&E staining revealed evidence of progressive or chronic fibrosis in all cases (100%), while hyperplasia of neurothelial cells was present in 60% of the cases (n=3) and dystrophic calcifications in 20% (n=1) (Figure 3). Immunohistochemical analysis using monoclonal antibodies against S100 and CD20 revealed that 60% of the samples (n=3) were positive for each marker. Immunohistochemical results were not correlated with any specific findings of H&E staining (Table 2). However, all these results suggest that the arachnoid membrane in patients with TN with CCP is responding to chronic inflammatory stimuli.

**Table 2:** Result of histochemical analysis of arachnoid biopsies CF - chronic fibrosis; HN - hyperplasia of neurothelial cells; DC - dystrophic calcification

Patient	CF	HN	DC	CD20	S100
1	+	+	-	+	+
2	+	+	+	+	-
3	-	+	-	+	+
4	+	-	-	-	-
5	+	-	-	-	+

## Discussion

Adhesive arachnoiditis is a non-specific and progressive inflammatory condition involving the leptomeninges and intrathecal neural elements [10]. The arachnoid membrane, the central membrane layer of meninges, is extremely thin and fragile. It lacks innervation or vascularization, making the healing process difficult. The mechanism of adhesive arachnoiditis is believed to be similar to adhesions formed during the injury repairment in other serous membranes lacking innervation or vascularization (e.g., peritoneal or pleural membrane). Due to the location of the arachnoid, the CSF removes and dilutes phagocytes and fibrinolytic enzymes, leaving the arachnoid unable to eliminate the fibrinous bands formed after a noxious stimulus. Furthermore, fibrocytes proliferate in these bands, leading to dense collagen deposition and encapsulation of the nerve roots, eventually resulting in progressive atrophy [11-14].

During MVD, we occasionally observe the presence of thickened and adhesive arachnoid membranes surrounding the nerve and neighboring vessels, resulting in anatomical alterations in the trigeminal cistern due to the coalescence of neurovascular structures. In some cases, as presented herein, the arachnoid becomes thick and firm enough to form taut-like bands that constrict the roots of CN V and/or the adjacent cranial nerves. Based on our experience, the thickened arachnoid membrane generally interferes with the correct observation of the surgical field and must be removed by careful microsurgical management. Caution must be exercised during this step of the surgery to avoid overstressing or causing a traumatic event in the trigeminal root or adjacent structures, which could lead to an undesirable outcome.

Although arachnoiditis is mainly described in the spine, it has been observed focally in the cranial cisterns [6,15,16]. Spinal arachnoiditis may be chemically or mechanically induced, either via iatrogenic origin or as a result of various infectious diseases, tumors, and genetic factors [17,18]. Chronic adhesive arachnoiditis is the most frequently mentioned term in the literature, representing the most severe type of arachnoiditis characterized by scar tissue compression of spinal nerve roots, leading to persistent pain [19]. Complete pain relief and the alleviation of symptoms remain unattainable in most cases of spinal arachnoiditis. Pharmacological treatment is primarily palliative, and surgical intervention remains controversial due to its challenging nature, the potential risks and poor outcomes reported [10,18]. Medication for reducing pain in spinal arachnoiditis patients may overlap with pharmacological treatment for TN, including opiates and anticonvulsants that act as central pain neuromodulators (e.g., gabapentin and phenytoin) [2,10]. Furthermore, patients suffering from spinal arachnoiditis may develop depression, drug dependence, and suicidal behavior as a result of years of persistent pain; tendencies frequently observed in patients with TN [2,10,18]. 

Determining the incidence and prevalence of arachnoiditis in the basal cisterns of the brain is particularly complicated due to the absence of specific symptoms, its distribution, and possible varied origin, making it difficult to develop a comprehensive clinical picture of this entity [10]. Furthermore, the classification of this type of arachnoiditis has not yet been standardized. It could be categorized based on its degree of severity, adherence to neurovascular structures and surrounding tissues, or based on characteristics such as thickening, opacity, or consistency; however, these approaches may result in subjective assessments. For practical purposes, and until a consensus is established, we have classified it as absent, mild, moderate, severe, or postsurgical. Based on our extensive surgical experience, all five cases presented here could be categorized as severe. Additionally, we have observed that it may exhibit two consistencies when manipulated: viscous or fibrous. The etiology behind the manifestation of these specific consistencies is also unclear.

A recent study analyzed changes in the CSF inflammatory profile using proximity extension assay (PEA) before and after MVD surgery and identified two proteins, members of the TNF superfamily, TNF-β, and TRAIL, that differed between patients with TN and all their control groups [5]. Both proteins are involved in demyelination, immune defense, and apoptosis. Although neuroinflammatory mechanisms may play an important role in the pathophysiology of TN, there are no reliable and harmless laboratory tests that demonstrate inflammation in the cisterns of the brain, leaving histopathological analysis as the most viable option to date to approach the study of this entity.

Further pathologic and histologic studies of thickened arachnoid membranes are needed. Therefore, we conducted a study on a selected group of patients with paroxysmal TN with CCP, who exhibited arachnoiditis findings during surgery. Microscopic histopathological findings in the biopsies of the arachnoid membrane, such as chronic fibrosis, hyperplasia of neurothelial cells, and dystrophic calcifications, are alterations commonly associated with the chronic inflammatory response [20-22]. Additionally, immunostaining revealed the involvement of S100, proteins associated with neoplasms and inflammatory disorders, and CD20, a transmembrane phosphoprotein that plays a role in B lymphocyte activation and differentiation [23,24]. All these results suggest a possible relation between chronic inflammation and the pathophysiology of TN, with arachnoiditis being a possible risk factor for developing paroxysmal TN with CCP.

Despite presenting a long-term follow-up, a limitation of this study was the small sample size; however, the results obtained suggest that further analyses are necessary, including patients with both clinical presentations of TN. This would help to identify specific characteristics in the altered arachnoid that may be associated with each type of TN. Furthermore, larger studies could help in classifying the severity of arachnoiditis based on histopathological findings rather than subjective observations.

## Conclusions

The origin of the altered arachnoid findings during MVD is not fully defined at the moment. Our results suggest that a chronic inflammatory process in the arachnoid accompanies paroxysmal TN with CCP, where fibrosis, dystrophic calcium deposition, and/or hyperplasia of neurothelial cells may occur secondary to a continuous noxious stimulus. Those insights could be related to a novel explanation for the etiology and pathophysiology of paroxysmal TN with CCP.
